# Comparison of scandium-44 g with other PET radionuclides in pre-clinical PET phantom imaging

**DOI:** 10.1186/s40658-019-0260-0

**Published:** 2019-12-12

**Authors:** Simon Ferguson, Hans-Sonke Jans, Melinda Wuest, Terence Riauka, Frank Wuest

**Affiliations:** Department of Oncology, University of Alberta, Edmonton, Canada

**Keywords:** Scandium, Image quality, PET imaging, Radiometals

## Abstract

**Purpose:**

The decay characteristics of radionuclides in PET studies can impact image reconstruction. ^44g^Sc has been the topic of recent research due to potential theranostic applications and is a promising radiometal for PET imaging. In this study, the reconstructed images from phantom measurements with scandium in a small-animal PET scanner are compared with ^18^F and two prominent radiometals: ^64^Cu and ^68^Ga

**Methods:**

Three phantoms filled with ^18^F, ^64^C, ^68^Ga, and ^44g^Sc were imaged in the Siemens Inveon PET scanner. The NEMA image quality phantom was used to determine the recovery coefficients (RCs), spill-over ratios (SORs), and noise (%SD) under typical pre-clinical imaging conditions. Image contrast was determined using a Derenzo phantom, while the coincidence characteristics were investigated using an NEC phantom. Three reconstruction algorithms were used, namely filtered back projection (FBP), ordered subset expectation maximization (OSEM), and maximum a-posteriori (MAP).

**Results:**

Image quality parameters were measured for ^18^F, ^64^Cu, ^68^Ga, and ^44g^Sc respectively; using FBP, the %SD are 5.65, 5.88, 7.28, and 7.70; the RCs for the 5-mm rod are 0.849, 1.01, 0.615, and 0.825; the SORs in water are 0.0473, 0.0595, 0.141, 0.0923; and the SORs in air are 0.0589, 0.0484, 0.0525, and 0.0509. The contrast measured in the 2.5-mm rods are 0.674, 0.637, 0.196, and 0.347. The NEC rate with ^44g^Sc increased at a slower rate than ^18^F and ^68^Ga as a function of activity in the field of view.

**Conclusion:**

^44g^Sc demonstrates intermediate behavior relative to ^18^F and ^68^Ga with regard to RC and contrast measurements. It is a promising radionuclide for preclinical imaging.

## Introduction

The physical properties of the radionuclide used in a PET study affect the quality of the reconstructed image and the quantification of reconstructed activity. The positron energy spectrum of the radionuclide determines the positron range. The positron range is one of the dominant factors affecting resolution, and this is especially pronounced in pre-clinical scanners with smaller crystal sizes [1, 2]. There have been many experimental and Monte Carlo studies investigating the positron range of radionuclides in various media [3–7]. Techniques for correcting the blurring caused by the positron range have been proposed [8, 9].

Some newly proposed radionuclides possess high-energy co-emitted gammas (i.e., prompt gammas) which negatively interfere with the detection of positron annihilation photon pairs [10]; added image noise, owing to the increased the rate of spurious coincidences, has been reported [11, 12] and is not directly accounted for in conventional PET corrections. The prompt gammas are often of different energy and therefore possess different scatter and detection kernels. Prompt gamma corrections have been proposed to increase the accuracy of quantitative imaging, but are often radionuclide specific and require further investigation before being applied to novel radionuclides [13–19].

^44g^Sc is of particular interest in PET imaging, and more broadly for theranostic applications in conjunction with ^47^Sc [20–23]. ^44g^Sc can be cheaply produced in high yields on low-energy cyclotrons through the proton irradiation of natural calcium or enriched ^44^Ca targets [24, 25]. ^44g^Sc can also be obtained through the decay of ^44^Ti; there have been initial developments into ^44^Ti/^44g^Sc generator systems [26–28]. Wider availability of the radionuclide has led to more radiolabelling and imaging studies [29–33]. However, the image quality and quantitative accuracy of ^44g^Sc needs to be investigated rigorously to determine its clinical relevancy with respect to other potential radiometals used for PET imaging.

Phantom imaging is a useful tool for comparing radionuclide performance under similar imaging conditions [34]. Thus far, phantom imaging with ^44g^Sc has been mainly limited to Derenzo phantoms. A radionuclide comparison in a Derenzo phantom was first reported by Bunka et al. comparing the relative spatial resolution of ^68^Ga, ^44g^Sc, ^89^Zr, ^11^C, ^64^Cu, and ^18^F [35]. Domnanich et al. later expanded on this study by comparing the resolution of Derenzo phantom images for ^44g^Sc and ^43^Sc obtained in different ratios through different production routes, such as the proton irradiation of enriched ^46^Ti and ^43^Ca [36].

While spatial resolution as a function radionuclide is of interest, other performance metrics also necessitate quantification when evaluating new imaging radionuclides [37]. The NEMA guidelines have provided a standardized procedure for evaluating the performance of small-animal PET scanners (NEMA NU4-2008). These same procedures might be used to compare image parameters between different radionuclides on the same scanner; this was done by Disselhorst et al. to compare the recovery coefficients and spill-over ratios for ^18^F, ^68^Ga, ^124^I, and ^89^Zr [38].

In this study, the imaging properties of ^44g^Sc are further assessed through phantom imaging in the Siemens Inveon small-animal PET scanner; this includes measurements of noise (%SD) and activity quantification (recovery coefficient (RC), spill-over ratio (SOR)) that were not previously determined. Three different phantoms are used to acquire relevant measurements. In addition, all quantitative parameters evaluated for ^44g^Sc were also measured for ^18^F, the most common PET radionuclide, as well as the two widely used PET radiometals ^68^Ga and ^64^Cu to allow intercomparison of all radionuclides.

## Materials and methods

### Radionuclides

Four radionuclides were used in this comparison study: ^18^F, the most commonly used PET radionuclide, and three radiometals, ^68^Ga, ^44g^Sc, and ^64^Cu.

*Fluorine-18*: The radiofluorine was produced locally at the Cross Cancer Institute (CCI) from enriched ^18^O water. With a half-life of 109.8 min, ^18^F is a pure, low-energy positron emitter, with an average and maximum energy of emission of 249.8 keV and 633.5 keV respectively (96.7% abundance).

*Copper-64*: Radiocopper was produced at Washington University in St. Louis, USA, and shipped to the University of Alberta in Edmonton, Canada. ^64^Cu has a 12.7 h half-life and decays through both β− (38.5 %) and β+ (17.6 %) decay. The positron is emitted with an average and endpoint energy of 278.2 and 653.0 keV respectively.

*Gallium-68*: Radiogallium was obtained from a ^68^Ge/^68^Ga generator (iThemba Laboratories, Sommerset West, South Africa). ^68^Ga decays with the shortest half-life of the radionuclides in this study, at 67.71 min. On the other hand, it emits the positron with the greatest average and endpoint energies, at 836.0 and 1890 keV respectively with 87.7% abundance and a total positron branching ratio of 88.8%. An additional 1077 keV gamma is emitted with 3.22% of decays.

*Scandium-44 g*: ^44g^Sc has a 3.97 h half-life and emits a 1157 keV gamma (99.9% abundance) in addition to a positron (94.27% abundance) with an average and maximum energy of 632.0 and 1474 keV respectively. The radioscandium was produced locally at the CCI through the irradiation of natural calcium with 16 MeV protons. The radionuclidic purity is reported in Table 1.

**Table 1:** Radioisotopic composition of radioscandium from the irradiation of natural calcium targets with 16 MeV protons

Isotope	Half-life	Percent activity at EOB	Percent activity at 9.5 h post EOB
^44g^Sc	3.97 h	94.9	90.1
^43^Sc	3.89 h	3.6	3.2
^44m^Sc	58.61 h	0.5	2.1
^47^Sc	3.35 d	0.4	1.8
^48^Sc	43.67 h	0.6	2.7

### Image acquisition

The Siemens Inveon PET platform was used to perform imaging experiments. Its detector consists of lutetium oxyorthosilicate (LSO) crystals coupled through a light-guide to position sensitive photo-multiplier tubes. The LSO crystals are arranged in 16 detector blocks, each with 4 detectors axially which are divided into 20 × 20 crystal arrays. The ring diameter is 16.1 cm and the axial length 12.7 cm, with individual crystal sizes of 1.5 × 1.5 × 10 mm^3^. For the acquisitions, an energy window of 350–650 keV and a coincidence timing window of 3.432 μs were used.

Prior to injection into the phantoms, the radionuclide activity was measured in an Atomlab 400 dose calibrator (Biodex Medical Systems, NY, USA). Emission data was acquired in list mode, and the Inveon Acquisition Workplace (v. 1.5.0.28) was used to bin the data into sinograms and reconstruct the images. Images were reconstructed with three image reconstruction procedures available, using the default parameters. The reconstruction procedures were 2D FBP (Fourier rebinning, Nyquist cut-off 0.5), OSEM3D-MAP (2 OSEM3D iterations, 18 MAP iterations, 1.5-mm requested resolution), and OSEM2D (4 iterations).

### Image noise, spill-over ratio, and recovery coefficient

**Fig. 1:**
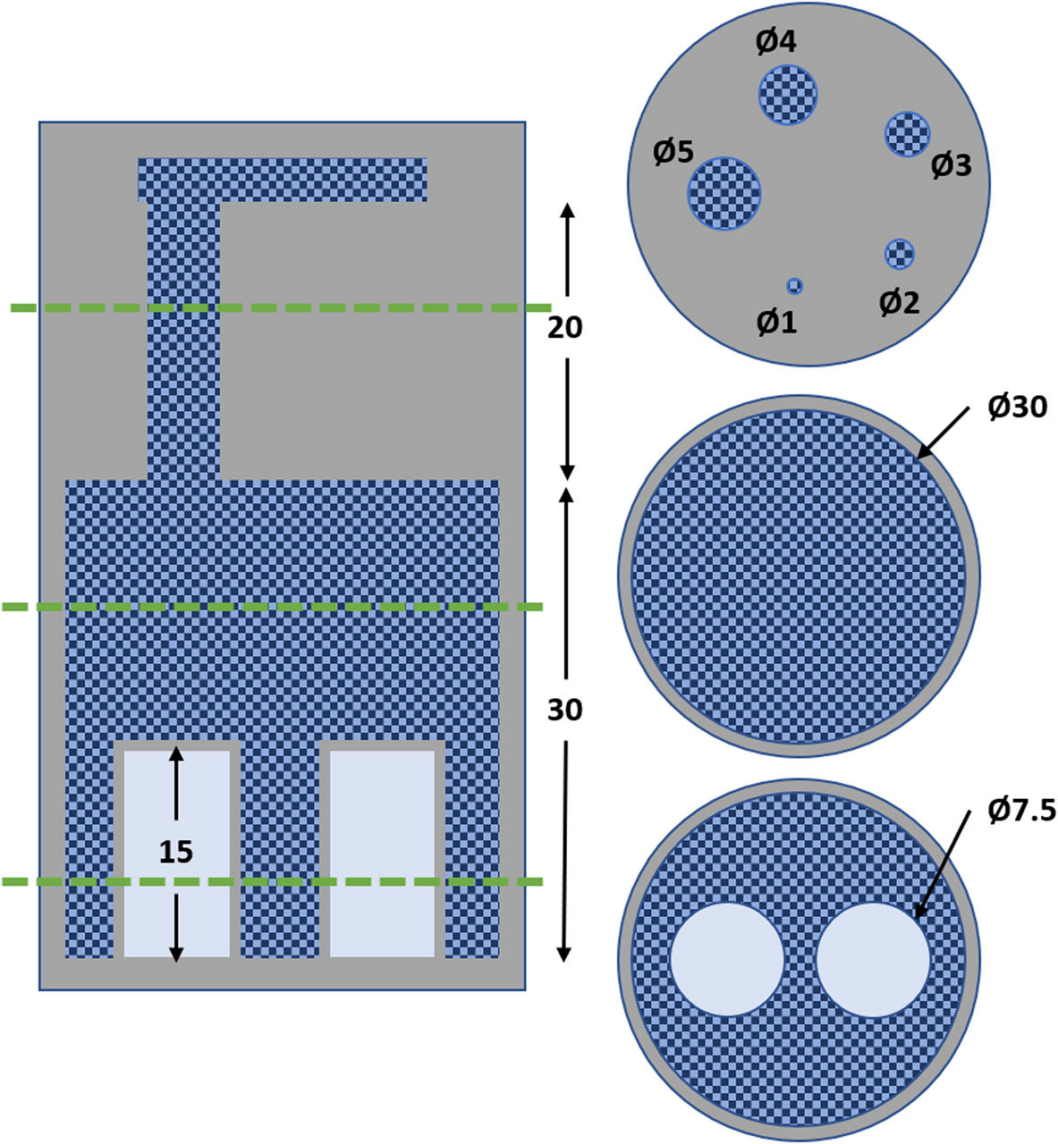
Cross-sections of the NEMA image quality phantom with dimensions in mm. Left: axial cross-section. Right: transverse cross-sections of the three different regions. The grey area represents the PMMA phantom; the dark blue hashed region represents the volume filled with activity, and the light blue represents the cold air and water volumes

As the quality of reconstructed images can vary under different imaging situations, the National Electrical Manufacturers Association (NEMA) has provided a standard for acquiring and evaluating the data equivalent to a full-body scan of a rodent with cold and hot regions (NEMA NU 4-2008). A NEMA image quality phantom consists of a polymethylmethacrylate (PMMA) cylinder with three distinct sections, as outlined in Fig. 1, with the fillable activity hashed in blue.

The NEMA guidelines state that measurements should be performed with a total activity of 3.7 MBq ± 5% of ^18^F and counts acquired over 1200 s. To compare standard metrics for different radionuclides, modifications to this procedure must be made. The number of positron decays for a given activity and time is affected by the radionuclide’s half-life and positron branching ratio. The approach taken in this study is to keep the starting activity 3.7 MBq and modify the acquisition time in order to achieve the same number of positron decays as ^18^F in 1200 s. Table 2 lists the number of positron decays for a 3.7 MBq source of each different radionuclide, as well as the acquisition time used to obtain the same number of counts as ^18^F in the guideline conditions.

**Table 2:** Number of decays expected in standard scan conditions and time used to acquire an equal amount of positron decays for each radionuclide.

Nuclide	Positron annihilations in 1200 s for 3.7 MBq	Time to acquire the same number of positron annihilations as ^18^F (s)
^18^F	4.03 × 10^9^	1200
^68^Ga	3.57 × 10^9^	1376
^64^Cu	0.774 × 10^9^	6506
^44g^Sc	4.07 × 10^9^	1190

After the emission acquisition, a 30-min transmission scan was acquired with a ^57^Co point source and used for attenuation correction. The reconstructed transmission image was segmented into five materials: background (0.00 cm^−1^), animal bed (0.0150 cm^−1^), water (0.095 cm^−1^), bone (0.1780 cm^−1^), and aluminum (0.22 cm^−1^).

To measure uniformity, the central uniform region of the phantom was contoured. A 22.5 mm diameter (75% of active diameter) and 10-mm-long cylindrical VOI were drawn, and the mean (*C*_uniform_), maximum and minimum values were recorded, while the percent standard deviation (*σ*_uniform_) was calculated.

The hot rod region was used to measure the recovery coefficient. The central 10 mm length of the rods were averaged, circular ROIs twice the size of each rod drawn, and the pixel with the maximum value in each ROI was found. This transverse pixel was used in an axial profile over the 10 mm to determine for each rod the mean (*C*_rod_) and standard deviation (*σ*_rod_). The recovery coefficient (*RC*) is the ratio of the mean value of the rods to that of the uniform region, while the uncertainty (*σ*_*RC*_) is calculated using the standard deviation.

1 $$ RC=\frac{C_{\mathrm{rod}}}{C_{\mathrm{uniform}}}\kern1em \mathrm{and}\kern1em {\sigma}_{RC}=100\ast \sqrt{{\left(\frac{ST{D}_{rod}}{C_{rod}}\right)}^2+{\left(\frac{ST{D}_{\mathrm{uniform}}}{C_{\mathrm{uniform}}}\right)}^2} $$

Finally, the spill-over ratio (SOR) in air and water was measured using a 4 mm diameter (50% of cylinder diameter) and 7.5-mm-long cylindrical volume for interest in the water- and air-filled inserts. The mean activity (*C*_cold_) and standard deviation (*STD*_cold_) were calculated in each ROI; the SOR is the ratio of the mean value in the inserts to the mean value of the uniform region, while the uncertainty (*σ*_*SOR*_) is calculated using the standard deviation.

2 $$ SOR=\frac{{\mathrm{C}}_{\mathrm{cold}}\ }{C_{\mathrm{uniform}}}\kern1em \mathrm{and}\kern1em {\sigma}_{SOR}=100\ast \sqrt{{\left(\frac{ST{D}_{cold}}{C_{cold}}\right)}^2+{\left(\frac{ST{D}_{uniform}}{C_{uniform}}\right)}^2} $$

### Contrast and feature size

**Fig. 2:**
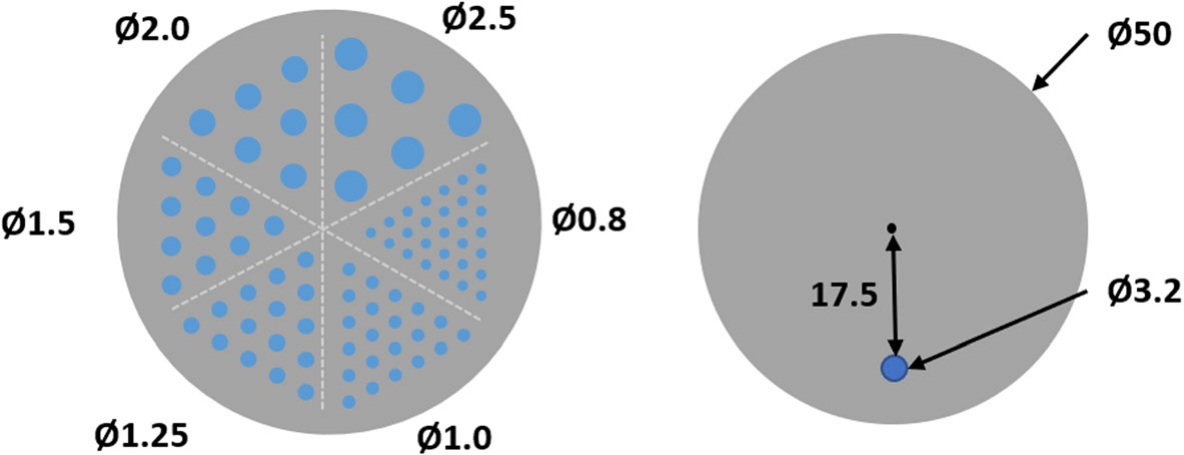
Phantom cross-sections with dimensions in mm. Left: cross-section of the Derenzo phantom with the fillable rod diameters. Right: NEC phantom cross-section

Regions of interest in pre-clinical scans can vary in size; many image quality models relate contrast to feature size and signal-to-noise ratio. It is important to understand the relationship between contrast and feature size because radionuclide properties, such as the positron energy spectrum, can affect lesion visibility and quantification. The Derenzo phantom is commonly used to quantify the trade-off between image contrast and visibility of small features. It is constructed from PMMA and contains triangular arrangements of hollow rods which can be filled with radioactivity. Each of the six sections contains rods of a given diameter (2.5, 2.0, 1.5, 1.25, 1.0, and 0.8 mm), and each rod is separated from its nearest neighbors by twice its diameter (center-to-center distance) as outlined in Fig. 2.

A large number of coincidences (greater than 5 × 10^7^) were acquired for each radionuclide in the Derenzo phantom, and the images were reconstructed with attenuation correction and a final reconstructed pixel size of 0.388 mm (zoom 2). A transverse slice was used to determine the contrast in each triangular region. A profile was drawn between the central pixels of the center-most rod and an outer rod. The peak (*C*_max_) and minimum (*C*_min_) values of this profile were utilized to calculate image contrast (*C*):

3 $$ C=\frac{C_{\mathrm{max}}-{C}_{\mathrm{min}}\ }{C_{\mathrm{max}}+{C}_{\mathrm{min}}} $$

### Coincidence characteristics

Scanner performance is affected by the amount of radioactivity and geometry of the object in the scanner’s field of view. Count losses occur as a result of camera dead time, decreasing the scanner’s counting rate capability. Additionally, some scanners manifest a difference in sensitivity to scattered and primary radiation [39]. In this study, following NEMA NU 4-2008, these effects are investigated for each radionuclide using a “rat”-sized phantom made of high-density polyethylene (density 0.96 ± 0.1 g/cm^3^) with 50 ± 0.5 mm diameter and a length of 150 ± 0.5 mm. A 3.2 mm diameter hole 17.5 mm from the center extends through the length of the phantom, through which a 140-mm line source containing the radionuclide of interest is inserted.

For this study, sources of ^18^F, ^44g^Sc, and ^68^Ga with activities greater than 100 MBq were placed in the phantom which was centered in the field of view, and counts were acquired over several half-lives as the radionuclides decayed. This study was not performed with ^64^Cu due to the low branching ratio and long half-life; the activity required to observe count rates similar to the other radionuclides would be significantly larger (5.5 times the activity of ^18^F to achieve the same amount of emitted positrons) and the acquisition time would greatly increase (greater than 3 days to decay from 100 to 1.5 MBq). Prior to binning into sinograms, the list-mode data was separated into 15-min time frames for ^18^F and ^68^Ga, and 30 min time frames for ^44g^Sc. These durations were chosen to be less than a quarter of each radionuclide’s half-life.

Single-slice rebinning was used to collapse oblique sinograms into single sinograms for each slice, with a span of 79 and ring difference of 39. No corrections were applied to the acquired counts, and the random coincidences were estimated in a separate sinogram. True (*R*_*T*_), random (*R*_*R*_), and scatter (*R*_*S*_) event rates, as prescribed by NEMA NU-4, were used to calculate the noise equivalent count rate, NECR, using:

4 $$ NECR=\frac{R_T^2}{R_T+{R}_S+{R}_R} $$

The NECR is the true count rate that would lead to the same amount of noise due to counting statistics in the absence of scattered and random coincidences

## Results

Measured results for each parameter are discussed below; summary of key numeric values for each parameter is provided in Table 3.

**Table 3:** Results from phantom imaging studies. The values reflect the FBP reconstruction for the NEMA Image quality phantom and Derenzo phantom studies

Nuclide	Half-life	Mean (max) positron emission energy (keV)	RC (5 mm)	%SD	SOR air	SOR water	Contrast	NECR at 10 MBq (kcps)
^18^F	109.8 m	249.8 (633.5)	0.849	5.65	0.0473	0.0589	0.674	132
^64^Cu	12.7 h	278.2 (654.0)	1.01	5.88	0.0595	0.0484	0.637	-
^44g^Sc	3.97 h	632.0 (1474)	0.825	7.70	0.0923	0.0509	0.347	127
^68^Ga	67.7 m	836.0 (1890)	0.615	7.28	0.141	0.0525	0.196	131

### Image noise, spill-over ratio, and recovery coefficient

**Fig. 3:**
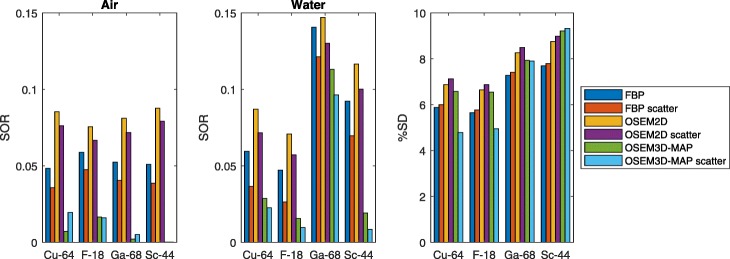
Impact of radionuclide and reconstruction algorithm on %SD and SOR in air and water. All data were acquired with the same number of counts. Both the uncorrected and scatter corrected values are presented for comparison

The percentage standard deviation (%SD) is a measure for noise in the reconstructed image, and the measured values are shown in Fig. 3 (rightmost chart). Its variation is similar for all radionuclides with FBP and OSEM2D reconstruction algorithms; however, OSEM3D-MAP with scatter correction increases noise for ^68^Ga and ^44g^Sc while decreasing it for ^18^F and ^64^Cu. While ^18^F and ^64^Cu exhibit similar values, %SD for ^68^Ga and ^44g^Sc is slightly increased. Note that the same number of counts was acquired for each radionuclide; an increase in %SD therefore indicates a relative decrease in the signal-to-noise ratio in the resulting image.

The spill-over ratio (SOR) is a measure for activity falsely assigned to regions in the reconstructed image in which no radioactivity was present during image acquisition. The SOR in air and in water are also found in Fig. 3 as a function of reconstruction algorithm and radionuclide. As expected, the scatter correction decreases the SOR because of the decrease of accepted scattered photons. The reconstruction strategy is the main determinant of the SOR in air which is largely independent of the radionuclide species, as demonstrated in Fig. 3.

The SOR in water has two distinct groupings: the SOR in water for short-range positron emitters (^18^F and ^64^Cu) is significantly smaller than for the long-range positron emitters ^44g^Sc and ^68^Ga. Contrary to air, the SOR in water is determined less by the scatter correction strategy than the positron range. Overall, SOR in water scales with positron range, with ^44g^Sc exhibiting intermediate values between ^64^Cu and ^68^Ga.

**Fig. 4:**
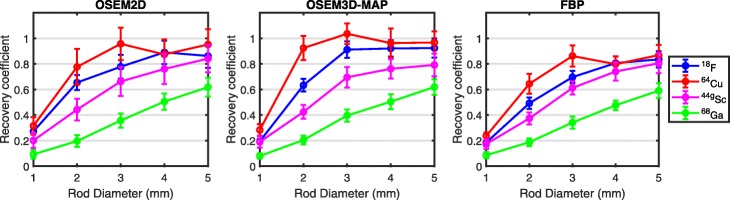
Impact of radionuclide and reconstruction strategy on measured recovery coefficients (RC)

The recovery coefficient (RC) is a measure of the fraction of activity reconstructed in a small region. RCs are plotted in Fig. 4 as a function of rod size for the different radionuclides and reconstruction strategies. The same overall trend is observed in all graphs: the RC increases towards unity with increasing rod diameter. The RC for ^64^Cu remains almost constant down to rod sizes of 2 mm before sharply dropping for the 1-mm rod. Among all isotopes, the RC for ^68^Ga is smallest for all rod sizes, while the RC for ^44g^Sc is only slightly below ^18^F, with a gradual decrease as a function of rod diameter.

### Contrast and feature size

**Fig. 5:**
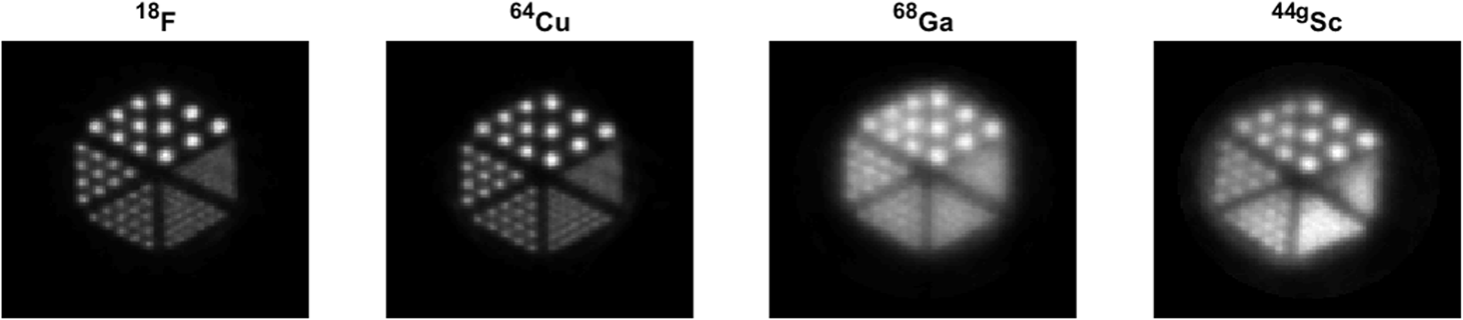
Derenzo phantom image reconstructed with OSEM2D for different radionuclides

A comparison of the reconstructed images acquired in the Derenzo phantom is shown in Fig. 5. The transverse cross-section of the hot rods demonstrates a clear difference in appearance between the short-range positron emitters ^18^F and ^64^Cu and the long-range positron emitters ^68^Ga and ^44g^Sc. For the latter two, the ability to distinguish smaller rods and separate them in the reconstructed images was impaired due to significant blurring.

**Fig. 6:**
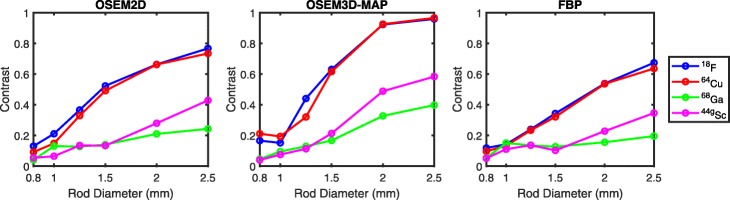
Normalized contrast as a function of rod size

The contrast between the rods and background in each of the six triangular segments was calculated (Eq. 2) and is shown in Fig. 6. There is clear separation between short-range positron emitters ^18^F and ^64^Cu, which demonstrate a contrast greater than 0.5 for feature sizes of 1.5 mm and above. While contrast of ^44g^Sc is superior to ^68^Ga for the larger rod diameters, both radionuclides show the same contrast for rod diameters of 1.5 mm and below, leading to a significant blurring of smaller features (Fig. 5). The rods can no longer be distinguished when their diameters shrink below 1 mm for the long-range positron emitters and 0.8 mm for the short-range positron emitters. This blurring is due to the extrinsic scanner resolution, which is significantly impacted by the positron range. While the contrast is expected to reach zero, measurements for the smallest rod diameters show non-zero values, owing to image noise. As there is a constant activity concentration, less counts are originating from the smaller rods, leading to increased noise; this increased noise can affect contrast measurements, as is apparent in OSEM3D-MAP measurements of contrast with respect to ^64^Cu and ^18^F in the 1.25-mm rod, in which ^64^Cu has a lower value. The 1.25-mm rod is in the regime in which the contrast is decreasing at the greatest rate for OSEM3D-MAP, and increased noise from the smaller diameter rod can affect measurements.

### Coincidence characteristics

**Fig. 7:**
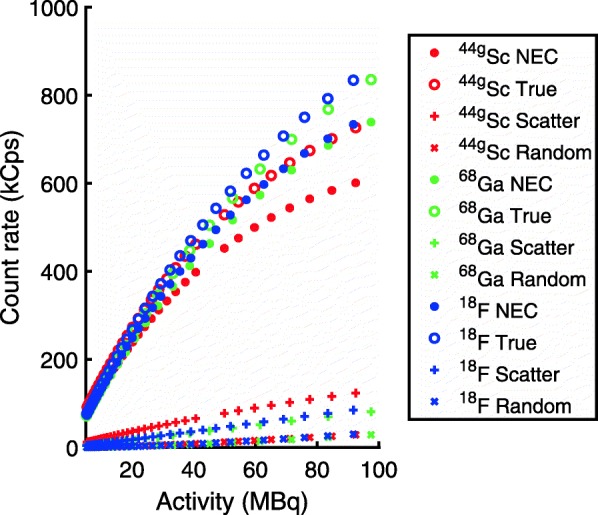
Event count rates as a function of radionuclide activity

The true, scatter, and random event rates measured using the NEC phantom are plotted in Fig. 7 along with the calculated NEC rate (Eq. 3). The random coincidence rate is expected to increase with the square of the single-photon rate, represented by the x-axis (activity); it was found that a second-order polynomial fits the random rate data for all isotopes in the range up to 100 MBq with an R-squared value of 0.999 as expected. The scatter contribution measured for ^44g^Sc is increased relative to ^18^F and ^68^Ga, while the true event count rate is decreased.

## Discussion

Imaging of the four radionuclides in the NEMA image quality phantom allowed for a comparison of ^18^F and the three radiometals ^64^Cu, ^44g^Sc, and ^68^Ga, in order of descending half-life and increasing positron range. The %SD measurements revealed a slight noise increase in images acquired with ^68^Ga and ^44g^Sc, although still within 2–3% of the ^18^F and ^64^Cu measurements.

The SOR measurements in water were largely affected by positrons annihilating in the cold volume, as previously observed by Disselhorst et al. in their comparison of the short-range positron emitters ^18^F and ^89^Zr in contrast with the long-range positron emitters ^68^Ga and ^124^I [38]. ^44g^Sc showed intermediate behavior as expected from a radionuclide with a mean positron emission energy of 632 keV, compared with ^18^F (250 keV) and ^68^Ga (836 keV). Overall, SOR in air is markedly decreased for the long-range positron emitters compared with water due to the greatly reduced electron density. However, the radius of the cold region would ideally exceed the maximum positron range of the radionuclide being investigated. Therefore, to obtain a true measure of the contribution of scatter and random coincidences to the reconstructed activity of cold regions, a different phantom design with a larger cold volume would be required for accurate measurements with long-range positron emitters; such a phantom, however, is not currently part of the NEMA test protocol.

The RC measurements revealed that activity recovery in 1 mm diameter structures is at 25% or less for all radionuclides and reconstruction algorithms and increases for larger structures. Once again ^44g^Sc exhibits intermediate behavior between ^18^F (short range) and ^68^Ga (long range). This indicates that a distinction beyond long-range and short-range positron emitters must be made when dealing with radionuclides with medium positron emission energies and that general trends can be predicted using positron emission energies.

Generally, the RC measurements for ^18^F and ^64^Cu agreed within the bounds of uncertainty. However, certain measurements show deviations, which are due to distinct image artifacts in which the activities in the center of the hot rods were underestimated and the edges were overestimated; these artifacts, known as the Gibbs phenomenon [40], lead to noisier measurements of RC and caused values to exceed the theoretical maximum of 1. The RC measurements are prone to large uncertainty as a single-pixel per transverse cross-section is averaged over an axial profile, and Gibb’s phenomenon will affect the central rod pixel value for different rod sizes; another challenge affecting the measurements is the alignment of the rods with the reconstructed voxel positions. As seen in Fig. 4, this phenomenon leads to noisier measurements for ^64^Cu and ^18^F; the RC of the 4-mm rod with ^64^Cu is measured as decreased compared with 3 mm and 5 mm but remains within the bounds of uncertainty, while the same applies to the RC of the 5-mm rod with ^18^F reconstructed with OSEM2D demonstrates a decrease with respect to the 4-mm rod. The Gibbs effect should be carefully considered when considering quantification in images reconstructed from activity distributions with sharp transitions, as is the case with hot rods and the short-range positron emitters ^64^Cu and ^18^F.

The measurements in the Derenzo phantom allow us to rank the relative contrast in the reconstructed images with the four radionuclides. We find similar measurements for the short-range positron emitters ^18^F and ^64^Cu, while the contrast is degraded with ^44g^Sc and to a greater extent with ^68^Ga for all reconstruction methods. The decreasing contrast can be predicted by the increasing positron energies as well as positron range, and follows the same trend as the relative resolution as determined by Bunka et al. to be ^18^F > ^64^Cu > ^44g^Sc > ^68^Ga [35].

The NEMA image quality study was conducted at activity levels commonly used for pre-clinical research; however, from the count rate curves acquired in the NEC phantom, the NECR curve for ^44g^Sc increases at a lesser rate than that of ^18^F. This indicates that with increasing activity, the noise is expected to increase at a greater rate for ^44g^Sc than ^18^F, which is likely due to spurious coincidences caused by the co-emitted 1.157 MeV gamma with 99% abundance. While this noise scaling is not a practical concern for pre-clinical investigations, it suggests that radionuclide specific image quality assessments could be beneficial when larger activities are present in the PET field of view. The total activity used in our studies with the NEMA image quality phantom was 3.7 MBq, which is representative of typical activities used for mice imaging; the %SD is expected to decrease, and the RC is expected to improve if the activity is increased because more counts are acquired with higher activities (assuming similar imaging times), while the SOR should remain constant or decrease as the count rates are in the linear regime at least for activities less than 20 MBq.

The NEMA NU 4-2008 standard for calculating event count rates does not address the case of spurious coincidences caused by the co-emission of prompt gammas during the radionuclide decay. Scattered and random events, which result from two annihilation photons detected in the energy window around 511 keV, cannot be distinguished from spurious coincidences, which involve the detection of at least one prompt gamma. At the current time, the impact of the contribution of spurious coincidences to the event counts for a radionuclide emitting prompt gammas must be assumed via a comparison with a pure positron emitter such as ^18^F.

Various methods to produce ^44g^Sc exist, each leading to a different radioisotopic composition of the scandium used for imaging. The radioscandium used in this study was produced from natural calcium targets with a radioisotopic purity of 95% ^44g^Sc at end of the beam and it remains at greater than 90% for 9.5 h afterwards (Table 1). Our most abundant co-produced isotope is ^43^Sc, which is a positron emitter as well and has a 3.89 h half-life. It is also considered a good candidate for PET imaging and has a favorable property that the most energetic and abundant positron emission has mean and maximum energy of 508 and 1199 keV respectively, which is lower than for ^44g^Sc. Consequently, its smaller positron range provides improved resolution compared with ^44g^Sc and ^68^Ga, as demonstrated by Domnanich et al. [36]. ^43^Sc may also be a more favorable choice for clinical studies from a radiation safety perspective because high-energy photon emissions, such as the 1157 keV prompt gamma (99.9% yield) emitted by ^44g^Sc, do not occur. Shielding for the high-energy photons emitted by ^44g^Sc does not pose significant challenge in pre-clinical studies (such as this one) because of the relatively low amounts of activity handled and the spatially confined nature of the experiments. When transitioning to patients, however, radiation safety aspects need to be carefully considered in order to ensure adequate protection of personnel and the public. ^43^Sc might then be preferable despite the somewhat more costly production process which utilizes an enriched calcium target. Initial in vivo human patient studies are underway to compare the dosimetric impact of ^44g^Sc on the patient [41]. Other co-produced radioisotopes with a total abundance of less than 1%, are ^44m^Sc (*t*_1/2_ = 58.61 h) which decays to ^44g^Sc and therefore also contributes to the total number of positrons available for imaging, as well as the two long-lived β- contaminants ^47^Sc (*t*_1/2_ = 3.35 days) and ^48^Sc (*t*_1/2_ = 43.67 h). These isotopes are not expected to significantly impact the imaging performance but may contribute to a small error in the absolute measurements of source activity. However, as they make up less than 1% of the total activity at end-of-beam (EOB), their impact is neither noticeable in images nor measurable within the uncertainties inherent in activity measurement with a radionuclide dose calibrator.

## Conclusions

The performance of the Siemens Inveon PET scanner was evaluated for ^18^F and the radiometals ^44g^Sc, ^64^Cu, and ^68^Ga. The most significant differences observed in our measurements can be attributed to the radionuclides’ positron emission energy: the short-range positron emitters ^18^F and ^64^Cu displayed greater recovery coefficients and contrast, as well as lower spill-over ratios than the long-range positron emitters ^68^Ga and ^44g^Sc. For a given radionuclide, the OSEM3D-MAP reconstruction provided the best contrast in the reconstructed images.

In conclusion, ^44g^Sc is a promising radionuclide for further study, as its intermediate positron emission energy provides increase contrast compared with ^68^Ga, another popular radiometal. The contribution of the high-energy gamma emission to image noise should be further studied because of its potential impact on image reconstruction when higher activity levels are present in the scanner’s field of view.

## Data Availability

The datasets used and/or analyzed during the current study are available from the corresponding author on reasonable request.
